# Archaeal origin of tubulin

**DOI:** 10.1186/1745-6150-7-10

**Published:** 2012-03-29

**Authors:** Natalya Yutin, Eugene V Koonin

**Affiliations:** 1 National Center for Biotechnology Information, National Library of Medicine. National Institutes of Health, Bethesda, MD 20894, USA

## Abstract

Tubulins are a family of GTPases that are key components of the cytoskeleton in all eukaryotes and are distantly related to the FtsZ GTPase that is involved in cell division in most bacteria and many archaea. Among prokaryotes, bona fide tubulins have been identified only in bacteria of the genus *Prosthecobacter*. These bacterial tubulin genes appear to have been horizontally transferred from eukaryotes. Here we describe tubulins encoded in the genomes of thaumarchaeota of the genus *Nitrosoarchaeum *that we denote artubulins Phylogenetic analysis results are compatible with the origin of eukaryotic tubulins from artubulins. These findings expand the emerging picture of the origin of key components of eukaryotic functional systems from ancestral forms that are scattered among the extant archaea.

**Reviewers:**

This article was reviewed by Gáspár Jékely and J. Peter Gogarten.

## Findings

Tubulins comprise a distinct family of GTPases that are highly conserved among eukaryotes and are the major components of microtubules, an essential part of the eukaryotic cytoskeleton [1,2]. All eukaryotes encode multiple, paralogous tubulins that evolved through a series of gene duplications at early stages of eukaryote evolution as well as many subsequent, lineage-specific duplications [3]. Among prokaryotes, the only *bona fide *tubulins have been identified in several bacteria of the genus *Prosthecobacter *[4] in which they form microtubule-like sturctures closely resembling eukaryotic microtubulues [5]. The tubulins of Prosthecobacteria show high sequence and structural similarity to eukaryotic homologs, and given their extremely narrow distribution among prokaryotes, are thought to have evolved via horizontal transfer of a eukaryotic tubulin gene to an ancestor of this group of bacteria [6,7]. The great majority of bacteria and many Archaea encode the FtsZ protein which plays a central role in cell division of most bacteria and many archaea and is a prokaryotic homolog of tubulin [8]. Both FtsZ and tubulin undergo GTP- hydrolysis-dependent cycles of polymerization and depolymerization, and are mechanistically analogous [9,10]. However, FtsZ and tubulin share extremely weak sequence similarity, so that the homology has become apparent only through comparison of crystal structures of these proteins [11]. Recent progress in genome sequencing and comparative genomics has revealed numerous previously unrecognized members of the FtsZ-tubulin protein superfamily [12,13]. These proteins considerably expand the range of sequence divergence adoptable by the FtsZ-tubulin fold but none of them are candidates for the role of direct prokaryotic ancestors of tubulins. In the absence of such candidates, it is generally assumed that tubulin evolved from FtsZ at the onset of eukaryote evolution, and this evolution engendered extreme sequence divergence associated with the shift in function [14]. Here we describe bona fide tubulins encoded in two recently sequenced genomes of Thaumarchaeota. Phylogenetic analysis suggests that these archaeal tubulins could be the direct ancestors of eukaryotic tubulins, a conclusion that has general implications for the evolution of the key functional systems of the eukaryotic cell.

### Archaeal tubulins

In the course of a systematic search for archaeal homologs of signature eukaryote proteins, we found that the best archaeal BLAST hits for tubulins were two closely related proteins from the recently sequenced genomes of Thaumarchaeota, *Candidatus *Nitrosoarchaeum limnia [15] and *Candidatus *Nitrosoarchaeum koreensis [16]. Eukaryotic tubulin sequences, in particular those of gamma-tubulins, aligned with these proteins over a region of approximately 300 amino acid residues, with e-values below 10^-13^. Although the similarity between eukaryotic tubulins and FtsZ-like proteins from other archaea were also statistically significant, these alignments only covered regions of approximately 100 amino acid centered at the GTP-binding loop, with most significant e-values of approximately 10^-8^. Reciprocal BLAST searches using the *Nitrosoarchaeum *tubulin homologs (hereinafter artubulins) as queries showed significantly greater similarity to eukaryotic tubulins than to FtsZ proteins.

**Figure 1:**
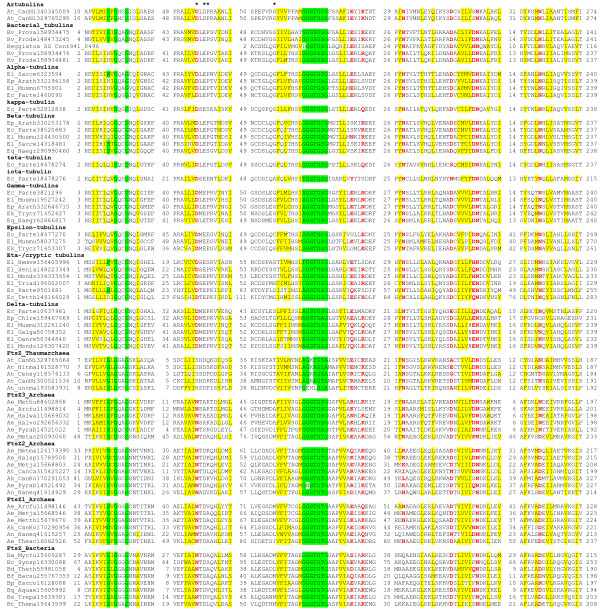
**Conserved sequence blocks in the tubulin/FtsZ superfamily**. The conserved blocks are separated by numbers which indicate the length of less well conserved sequence segments that are not shown (see Additional File 1). The alignment columns are colored on the basis of the respective position conservation throughout the superfamily: yellow background indicates hydrophobic residues (ACFILMVWY), red letters indicate polar residues (DEHKNQR), and green background indicates small residues (ACGNPSTV). Asterisks indicate amino acid residues that are conserved in the majority of the tubulins including artubulins but not in the majority of the FtsZ sequences. Each sequence is denoted by the corresponding taxon abbreviation followed by the species abbreviation and GenBank Identification (GI) number. Taxa abbreviations: A, Archaea; B, Bacteria; E, Eukaryota; Ac, Crenarchaeota; Ae, Euryarchaeota; An, Nanoarchaeota; At, Thaumarchaeota; Bv, *Verrucomicrobia*; Ec, *Alveolata*; Ek, *Euglenozoa*; El, *Fungi*/*Metazoa *group; Ep, *Viridiplantae*; Eq, *Heterolobosea*. Species abbreviations: Arath, *Arabidopsis thaliana*; Chlre, *Chlamydomonas reinhardtii*; Danre, *Danio rerio*; Drome, *Drosophila melanogaster*; Galga, *Gallus gallus*; Mondo, *Monodelphis domestica*; Musmu, *Mus musculus*; Naegr, *Naegleria gruberi*; Naneq, *Nanoarchaeum equitans *Kin4-M; Nemve, *Nematostella vectensis*; Ornan, *Ornithorhynchus anatinus*; Parte, *Paramecium tetraurelia*; Phypa, *Physcomitrella patens*; Plakn, *Plasmodium knowlesi *strain H; Plavi, *Plasmodium vivax *SaI-1; Prodeb, *Prosthecobacter debontii*; Prodej, *Prosthecobacter dejongeii*; Prova, *Prosthecobacter vanneervenii*; Sacce, *Saccharomyces cerevisiae *S288c; Strpu, *Strongylocentrotus purpuratus*; Tetth, *Tetrahymena thermophila*; Triad, *Trichoplax adhaerens*; Trycr, *Trypanosoma cruzi*; Xenla, *Xenopus laevis*.

These observations prompted us to perform a detailed phylogenetic analysis of the tubulin protein family. To this end, we compiled a representative set of eukaryotic and bacterial tubulins and constructed a multiple alignment of the sequences of these proteins with the artubulins (Figure 1; see Additional File 1 for the complete alignment). Examination of the conserved sequence motifs in the tubulin/FtsZ superfamily reveals several amino acid residues that are common to the tubulin family including artubulins but to the exclusion of FtsZ (Figure 1). The presence of the apparent synapomorphies is best compatible with a common origin of artubulins and the rest of the tubulin family.

**Figure 2:**
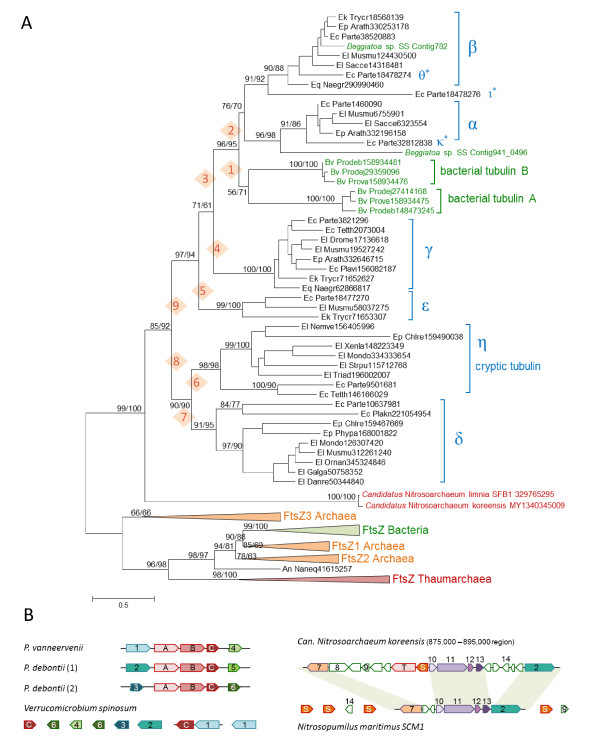
**Archaeal, bacterial and eukaryotic tubulins**. (A) Phylogenetic tree of the FtsZ-tubulin superfamily. The tree is rooted by FtsZ proteins; TreeFinder/Molphy bootstrap values are indicated for major branches. The sequences are denoted as in Figure 1. Asterisks mark diverged ciliate tubulins [12]. (B) Genome neighborhood of bacterial and thaumarchaeal tubulins. Genes are marked as follows: A, bacterial tubulin A; B, bacterial tubulin B; C, tetratricopeptide repeat protein referred to as bacterial kinesin light chain in ref (PMIDS 12486237 and 17942428); T, thaumarchaeal tubulin; S, Snf7; 1, putative serine/threonine kinase; 2, pyruvate phosphate dikinase; 3, aspartate aminotransferase; 4, response regulator with a HTH DNA-binding domain; 5, glucose/sorbose dehydrogenase; 6, cysteine synthase; 7, DEAD/DEAH box helicase; 8, Major facilitator superfamily MFS 1; 9, TATA-box binding protein; 10, zinc-binding CMP/dCMP deaminase; 11, DNA polymerase I; 12, conserved hypothetical protein; 13, triosephosphate isomerase; and 14, AsnC family transcriptional regulator. Syntenic regions between *Nitrosarachaeum koreensis *and *Nitrosopumilis maritimus *are shaded.

The multiple alignment of the tubulin/FtsZ superfamily (see Additional File 2) was employed to build maximum likelihood phylogenetic trees using FtsZ proteins as the outgroup. In the resulting phylogenetic tree, the artubulins form the sister group to all eukaryotic and bacterial tubulins (Figure 2A). In contrast, the *Prosthecobacter *tubulins were the sister group of the eukaryotic alpha/beta tubulin branch. Furthermore, this branch included two distinct tubulins that we identified in partial genomic sequences of the giant gamma proteobacterium *Beggiatoa *(Figure 2A). Thus, all available bacterial tubulin sequences grouped within the eukaryotic tubulin family. Constrained tree analysis showed that alternative tree topology, in which the artubulins grouped with bacterial tubulins, could be rejected at a statistically significant level; grouping of artubulins with different families of eukaryotic tubulins could not be similarly rejected (with one exception) although all alternative topologies showed lower likelihood than the tree in Figure 2A (see Additional File 3). These findings appear to be best compatible with a scenario in which the artubulins are direct evolutionary ancestors of the eukaryotic tubulins whereas bacterial tubulins originated as a result of horizontal transfer of eukaryotic alpha-beta tubulin genes into at least two bacterial lineages.

### Partially conserved genomic neighborhoods of archaeal and bacterial tubulin genes: functional and evolutionary implications

Notably, in the two *Nitrosarchaeum *genomes the tubulin gene is located next to the Snf7 gene which encodes one of the subunit of the ESCRT-III complex (Figure 1B). The ESCRT-III complex is conserved in all eukaryotes and is involved in intracellular membrane remodeling [17]. Recently, archaeal homologs of ESCRT-III subunits have been identified and shown to function as an essential component of the cell division apparatus in some archaea, primarily from the phyla Crenarchaeota and Thaumarchaeota [18-22]. In particular, it has been shown that *Nitrosopumilis maritimus*, a thaumarchaeon that belongs to the same family, *Nitrosopumillaceae*, as the tubulin-encoding *Nitrosoarchaeum*, employs ESCRT-III as the cell division machinery despite the presence of FtsZ [23]. In addition to artubulins, the two *Nitrosarchaeum *genomes encode regular FtsZ proteins that show very close sequence similarity to the *Nitrosopumilis *FtsZ (Figure 1). However, given the above data on the non-essentiality of FtsZ for cell division in *Nitrosopumilis *and the fact that artubulin and Snf7 genes are colocalized and possibly coexpressed in *Nitrosoarchaeum *(Figure 2B), it can be predicted that artubulin and ESCRT-III cooperate in cell division in these organisms.

The genomic neighborhood of artubulin-Snf7 in *Nitrosoarchaeum *has a readily detectable counterpart in *Nitrosopumilis*: a block of genes including those for artubulin and Snf7 appear to be inserted into the conserved neighborhood between the genes for a Superfamily 2 helicase and CMP/dCMP deaminase (Figure 2B). This relationship between the genome organizations of *Nitrosoarchaeum *and *Nitrosopumilis *suggests the divergence between artubulin-encoding and artubulin-lacking Thaumarchaeota involved rearrangement, possibly associated with horizontal gene transfer. The genes encoding bacterial tubulins are embedded in a completely different genomic neighborhood; however, a parallel exists with artubulins in that the tubulin genes in *Prosthecobacter *appear to have been inserted into a neighborhood that is partially conserved in distantly related *Verrucomicrobia *that lack tubulins (Figure 2B). Moreover, similarly to *Nitrosoarchaeum*, some *Prosthecobacter *genomes encode both tubulin and FtsZ [24].

### Implications for eukaryogenesis

Recent comparative genomic research on the origin of eukaryotes has revealed an unexpected pattern of archaeo-eukaryotic evolutionary relationship. Likely ancestors of the key functional systems of the eukaryotic cell have been shown to be scattered among diverse extant archaea. The cases in point include DNA polymerases [25], RNA polymerase subunits [26], various molecular complexes involved in membrane remodeling and cell division [20], and the ubiquitin signaling system [27]. The ubiquitin case appears particularly striking: a single archaeal genome, *Caldiarchaeum subterraneum*, that is likely to represent a distinct phylum [28], encompasses genes encoding all the essential components of the ubiquitin system [27]. The artubulins add another key piece to the puzzle of eukaryote origin. Although currently pertaining to a single archaeal lineage, this finding has substantial implications given that tubulins comprise one of the most abundant, highly conserved, essential protein families in eukaryotes. The likely origin of eukaryotic tubulins from ancestral forms represented in a specific lineage of archaea mimics the evolutionary scenario for actins, the second family of the major cytoskeletal protein in eukaryotes. The archaeal actin homologs, denoted crenactins, which are the probable ancestors of the eukaryotic actin family [29], are represented in *Thermoproteales*, *Korarchaeum cryptofilum*, and *C. subterraneum *[20,28], and form cytoskeletal elements essential for cell division in at least some of these Archaea [30]. It appears most likely that in *Nitrosoarchaeum*, which does not encode actin, a similar role belongs to artubulin. Taken together, these findings reinforce the mosaicism of the archaeal roots of eukaryotes and seem to be best compatible with the hypothesis that the archaeal ancestor of eukaryotes was a highly complex, possibly transient prokaryotic organism [31].

## Conclusions

We show here that two species of Thaumarchaeota from the genus *Nitrosoarchaeum *encode members of the FtsZ/tubulin superfamily that are more closely related to eukaryotic tubulins than to any archaeal or bacterial homologs; we denote these proteins artubulins. The results of phylogenetic analysis are compatible with the basal position of the artubulins in the tubulin family and hence with the ancestral status of artubulins with respect to the eukaryotic tubulins. This finding adds more weight to the shaping scenario of the origin of the first eukaryotic cells from highly complex, possibly transient archaeal forms.

## Methods

Protein sequences were retrieved from the non-redundant database at the National Center for Biotechnology Information (NIH, Bethesda). A reference set of FtsZ proteins was taken from a previous study [13]. Information on highly diverged eukaryotic tubulins was taken from [12,32]. The non-redundant protein sequence database was searched using the PSI-BLAST program [33]. Protein sequences were aligned using the MUSCLE program [34]; columns containing a large fraction of gaps (greater than 30%) and non-homogenous columns defined as described previously [35] were removed from the alignment. The resulting 278-column alignment was used to construct two maximum likelihood (ML) phylogenetic trees, one using the FastTree program [36] with default parameters (JTT evolutionary model, discrete gamma model with 20 rate categories) and the other using the MOLPHY program [37] with the JTT substitution matrix to perform local rearrangement of an original Fitch tree [38]. Phylogenetic tree topology testing was performed with the TreeFinder program [39] using either expected likelihood weights (ELWs [40]) or the approximately unbiased (AU) test *P *values [41]. An unconstrained ML tree was compared with 9 constrained topologies, which were constructed by grouping the artubulins with one of the branches major branches of tubulins (see Additional File 3).

## Competing interests

The authors declare that they have no competing interests.

## Authors' contributions

NY collected the data; NY and EVK analyzed the data; EVK wrote the manuscript which was read and approved by both authors.

## Reviewers' reports

### Reviewer 1: Gáspár Jékely, Max Planck Institute for Developmental Biology, Tuebingen, Germany

The identification of the *Nitrosoarchaeum *tubulins by Yutin and Koonin is potentially interesting, and upon first read I tended to agree with their conclusion that the results are compatible with the origin of eukaryotic tubulins from Nitrosoarchaeum tubulins. However, upon closer inspection, I found a few potential caveats with this interpretation, and I would like to ask the authors to address these. Additionally, I would also like to suggest a few points that would need further clarification.

The authors write that "Eukaryotic tubulin sequences ... aligned with these proteins [*Nitrosoarchaeum *tubulins] over a region of approximately 300 amino acid residues" and that "the similarity between eukaryotic tubulins and FtsZ-like proteins ... covered regions of approximately 100 amino acid centered at the GTP-binding loops". Having performed the blast searches, I can confirm these results. However, the statement like this is slightly misleading, since it implies that *Nitrosoarchaeum *tubulins are related to eukaryotic tubulins across 300 residues, and FtsZ only across 100 residues, and that is not true. If one performs psi-blast searches, after three iterations it becomes apparent that the alignments with *Nitrosoarchaeum *tubulins and FtsZ proteins all cover about 61-66% of the query sequence (I used mouse alpha tubulin as query). This section should be clarified to indicate that the region of homology is not longer between the Nitrosoarchaeum sequences and tubulins, than between FtsZ and tubulin.

Authors' response: *The text in question pertains to a single iteration of BLAST search and the observed differences are highlighted to emphasize the greater similarity between artubulins and tubulins compared to the tubulins versus FtsZ. There is no implication that the actual homologous domains are of different size. Indeed, it should be obvious the GTPase domain is conserved as a whole*.

I would like to point out a caveat about the rooting of the tree in Figure 2. The authors chose to root it on FtsZ proteins, however, with the same topology, the tree could also be rooted on the *Nitrosoarchaeum *sequences, and this would show the FtsZ clade as a sister to eukaryotic tubulins. Alternatively, the root could also be placed between eukaryotic sequences and FtsZ + *Nitrosoarchaeum*. These different rootings would dramatically affect the conclusions of the paper. The only justification for using FtsZ as a root, I assume, is that in blast searches the Nitrosoarchaeum sequences show higher similarity to the eukaryotic tubulins. This means, that the phylogenetic tree does not constitute independent evidence from blast, and therefore does not confirm the close relationship between the *Nitrosoarchaeum *sequences and eukaryotic tubulins.

Authors' response: *The rooting of the tree in *Figure 2*is justified not so much by sequence similarity but by phyletic distribution of tubulins and FtsZ. Indeed, both Nitrosoarchaea, in addition to the artubulins, encode FtsZ proteins typical of other Thaumarchaeota. Rooting the tree by artubulins would imply an ancient duplication with subsequent massive loss of artubulin genes in all bacteria and archaea except for two Nitrosoarchaea which is an extremely non-parsimonious scenario*.

The good blast score is due to alignments that are longer between the eukaryotic tubulins and the Nitrosoarchaeum sequences. However, if one looks at the extended alignment at the C-terminal side, the similarity is really poor, and this similarity is not picked up by blast, if only this portion is used. Could this extended alignment be due to residue composition or other bias (e.g. Nitrosoarchaeum sequences are less derived than FtsZ)? There is another disturbing observation. Namely, if one blasts with the portion of eukaryotic tubulins that are represented in the alignment (e.g. El_Musmu58037275), the best hit is to Thermococcus FtsZ (2e-08), and not Nitrosoarchaeum, that doesn't even show up until a psi-blast iteration is performed. Since the argument hinges on the phylogenetic tree, the above considerations should inspire extra caution.

Authors' response: *These concerns seem to stem from a certain misunderstanding of the way BLAST algorithm works. The algorithm extends the initial hit to the extent that is statistically justified and halts when further extension leads to increased E-values. Therefore a longer alignment recovered by BLAST is indeed evidence of greater sequence similarity. Spurious extension of an alignment due to compositional bias is possible but highly unlikely given the composition-based statistical corrections implemented in the current version of BLAST *[42,43].

As a minor comment, I suggest that the authors discuss in more detail the phylogenetic position of the Prosthecobacter tubulins in their tree. In particular, since it has been suggested by others that Prosthecobacter tubulins may by ancestral to all eukaryotic tubulins. For example, Pilhofer et al. [5] speculate about a "vertical evolution" scenario where eukaryotic tubulins evolved from the bacterial ones. This may have been justified given the poor resolution of their trees, showing no clear relationship between Prosthecobacter tubulins and any of the eukaryotic tubulin families. The present paper shows a tree (the first one to my knowledge) that finds strong support for a clade uniting Prosthecobacter tubulins with alpha and beta tubulins. This strongly argues against the vertical evolution scenario. This would be important to discuss, given that these bacterial tubulins sometimes feature in arguments about a purported evolutionary connection between eukaryotes and Planctomycetes-Verrucomicrobia-Chlamydiae bacteria, e.g. [44].

The relationship of *Prosthecobacter *tubulins and alpha and beta tubulins is not resolved. Others concluded [6] that *Prosthecobacter *tubulins have mosaic sequences with intertwining features from both alpha and beta tubulin. This analysis and the tree shown in this paper are consistent with a scenario where *Prosthecobacter *tubulins arose from an early horizontal gene transfer from an ancient tubulin, prior to the duplication of alpha and beta. This may be interesting to point out. It would also be interesting to see a technical comment on why the position of *Prosthecobacter *is resolved in the present tree, but not in previous attempts. Was there a difference in methodology? Were the sequence evolution models used more realistic in this study?

Authors' response: *Pinpointing the exact reasons behind differences in the results of phylogenetic analyses is very difficult. We are inclined to believe that the key factor is the more representative and balanced species sampling behind the trees presented here*.

*That said, we have investigated the phylogenetic position of bacterial tubulins in greater details, with the following conclusions*.

1. *Placing the bacterial branches outside the eukaryotic tubulin subtree was firmly rejected by the same statistical test of tree topology that we did in the paper (AU < 0.01). Thus, we have reasonable confidence that Prosthecobacterial tubulins are not ancestors to eukaryotic tubulins*.

2. *Monophyly of bacterial tubulins remains a matter of considerable uncertainty. This clade is not strongly supported in the tree in *Figure 2*(bootstrap value of 71 at best). Furthermore, per suggestion of reviewer 2, we ran ProtTest *[39]*to select the best substitution matrix which in this case turned out to be the LG matrix. Two alternative trees, using RAxML *[45]*and Treefinder *[39]*, were constructed from the same alignment as used for the tree shown in *Figure 2A. *In both trees, Prosthebacterial tubulins A and B grouped, respectively, with eukaryotic tubulins α and β but the respective branches were not supported (*Additional File 4*). In addition, eukaryotic and Prosthecobacterial tubulins were realigned without artubulins and FtsZ, in order to obtain an extended, higher quality alignment, and a tree was constructed using TreeFinder (*Additional File 4*). In this tree, bacterial tubulin A grouped with α/κ tubulins whereas bacterial tubulin B grouped with γ tubulins, exactly reproducing the topology in *Figure 6 *of Pilhofer *et al. [5]*but again with weak support*.

*Thus, we can only assert that Prosthecobacterial tubulins evolved from within the eukaryotic subtree but the actual scenario for their evolution remains uncertain. However, even this conclusion is sufficient to dismiss Prosthecobacterial tubulins as an argument for an evolutionary connection between the PVC superphylum of bacteria and eukaryotes. This connection seems to be non-existent as argued in detail elsewhere *[46].

### Reviewer 2: J. Peter Gogarten, University of Connecticut

The manuscript by Natalya Yutin and Eugene Koonin reports an exciting discovery: the presence of tubulin encoding genes in Thaumarchaeota. Tubulins are an important component of the eukaryotic cytoskeleton. The absence of closely related sequences ancestral to tubulins in prokaryotes was used to argue for a eukaryotic stem group that existed for a long period of time as a lineage distinct from archaea and bacteria. The argument is that a lot of substitutions would be needed to evolve tubulin from ftsZ, and that these many substitutions would be more compatible with a deep origin of the eukaryotes (see [47] for discussion). The finding of archaeal tubulins weakens this argument: If some archaea possess tubulins that branch outside the eukaryotic tubulins, but much closer to the tubulins than to FtsZ, then it is conceivable that the eukaryotes branch from inside the archaeal domain and inherited the tubulin from their archaeal ancestor.

Authors' response: *Indeed, the discovery of artubulins seems to invalidate the use of the distant relationship between tubulins and FtsZ as an argument for a eukaryotic stem outside Archaea. Along with other recent observations*, e.g. [20], *these findings seem to be best compatible with the origin of eukaryotes from a highly complex, possibly transiently existing archaeon *[29,31].

The archaeal tubulin sequences presented and discussed by Yutin and Koonin appear more similar to the eukaryotic tubulins than to FtsZ. This observation is confirmed by their phylogenetic analysis. The authors discuss their findings with appropriate caution, and I don't think that more sophisticated analyses will change the findings; nevertheless, the following two concerns seem worthy of consideration: First, the phylogenetic reconstruction does not appear to consider among site rate variation (ASRV), i.e., the choice of model used in phylogenetic reconstruction using maximum likelihood is not well described and justified. If one were to incorporate ASRV, I expect the deep branches of the phylogeny to become longer, because multiple substitutions are more efficiently corrected for, and the distinction between tubulins (including the archaeal tubulins) and FtsZs becomes stronger, strengthening the authors conclusion that these are indeed tubulins. Nevertheless, the choice of model should be discussed, and could be improved.

Authors' response: *We applied ProtTest and found LG to be the optimal matrix; accordingly, two alternative trees were built using LG (*Additional File 4*). The topologies of these trees differ in many places from the topology of the tree in *Figure 2*but these differences do not affect the conclusions of this work (see also the response to Reviewer 1 regarding the bacterial tubulins)*.

Second, aligning divergent sequences is difficult, and the alignment itself can create a strong phylogenetic bias. This is a concern, because the archaeal tubulins and the FtsZ sequences are very divergent. How certain can we be that these archaeal sequences group outside the eukaryotic domain, as one would expect if archaeal tubulins were ancestral to the eukaryotic ones, and not inside the eukaryotic domain, as one would expect if the Thaumarchaeota acquired the tubulins from a eukaryote through horizontal gene transfer. An analysis that simultaneously considers phylogeny and alignment, such as SATé [48], might help to exclude the possibility of a eukaryote to archaeon transfer with more confidence. However, the best approach to address this uncertainty will be additional archaeal tubulin sequences, which hopefully will become available in the future.

Authors' response: *SATé is beyond doubt an attractive phylogenetic approach but one that has not been sufficiently tested on phylogenies including distantly related, real sequences. We fully agree with the reviewer that the primary advance is likely to be brought about by further sampling of diverse archaea that is expected to reveal a greater diversity of artubulins*.

Minor suggestions: In spelling the species names for candidatus species, the convention is to italicize the word *Candidatus*, and to leave the suggested species name in normal font, e.g., *Candidatus *Nitrosoarchaeum koreensis. As no members of the genus have been cultivated, the *Candidatus *should also be used for the genus (e.g., the corresponding line in the abstract should read: "... genus *Candidatus *Nitrosoarchaeum that we denote artubulins. Phylogenetic ..." - also, the period was missing after artubulins). 1. Fournier GP, Dick AA, Williams D, Gogarten JP (2011) Evolution of the Archaea: emerging views on origins and phylogeny. Research in microbiology 162: 92-98. 2. Liu K, Raghavan S, Nelesen S, Linder CR, Warnow T (2009) Rapid and accurate large-scale coestimation of sequence alignments and phylogenetic trees. Science 324: 1561-1564.

Authors' response: *Corrected*.

## Supplementary Material

Additional file 1 **Multiple alignment of Tubulin/FtsZ family proteins**. Click here for file

Additional file 2 **Filtered multiple alignment of Tubulin/FtsZ family proteins used for tree construction**. Click here for file

Additional file 3 **Statistical tests on the topology of the phylogenetic tree of the tubulin/FtsZ superfamily**. Click here for file

Additional file 4 **Additional phylogenetic trees constructed using the RAxML and TreeFinder methods**. Click here for file
