# Supplementary material for: Archaeal origin of tubulin
Source: Biol Direct. 2012 Mar 29;7:10. doi: 10.1186/1745-6150-7-10 (PMC3349469; doi:10.1186/1745-6150-7-10)
Supplement: Additional file 3 — Statistical tests on the topology of the phylogenetic tree of the tubulin/FtsZ superfamily. [file 1745-6150-7-10-S3.PDF]

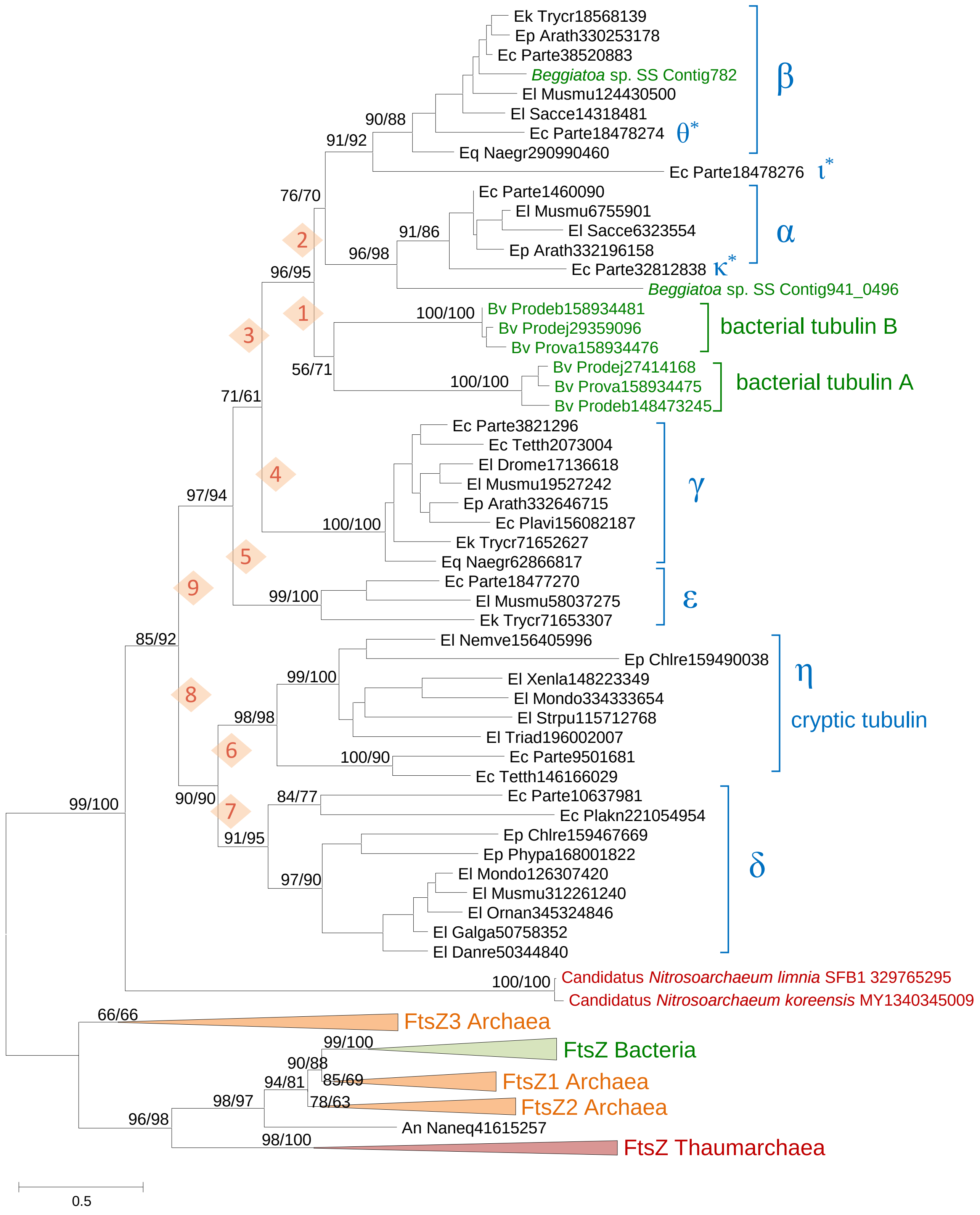
Ek Trycr18568139
Ep Arath330253178
Ec Parte38520883
Beggiatoa sp. SS Contig782
El Musmu124430500
El Sacce14318481
Ec Parte18478274
θ*
Eq Naegr290990460
Ec Parte18478276
ι*
β
90/88
91/92
76/70
Ec Parte1460090
El Musmu6755901
El Sacce6323554
Ep Arath332196158
Ec Parte32812838
κ*
α
91/86
96/98
Beggiatoa sp. SS Contig941_0496
96/95
Bv Prodeb158934481
Bv Prodej29359096
Bv Prova158934476
100/100
bacterial tubulin B
Bv Prodej27414168
Bv Prova158934475
Bv Prodeb148473245
100/100
bacterial tubulin A
56/71
71/61
Ec Parte3821296
Ec Tetth2073004
El Drome17136618
El Musmu19527242
Ep Arath332646715
Ec Plavi156082187
Ek Trycr71652627
Eq Naegr62866817
100/100
γ
97/94
Ec Parte18477270
El Musmu58037275
Ek Trycr71653307
99/100
ε
85/92
El Nemve156405996
Ep Chlre159490038
El Xenla148223349
El Mondo334333654
El Strpu115712768
El Triad196002007
Ec Parte9501681
Ec Tetth146166029
99/100
98/98
100/90
η
cryptic tubulin
90/90
Ec Parte10637981
Ec Plakn221054954
Ep Chlre159467669
Ep Phypa168001822
El Mondo126307420
El Musmu312261240
El Ornan345324846
El Galga50758352
El Danre50344840
84/77
91/95
97/90
δ
99/100
Candidatus *Nitrosoarchaeum limnia* SFB1 329765295
Candidatus *Nitrosoarchaeum koreensis* MY1340345009
100/100
66/66
FtsZ3 Archaea
99/100
FtsZ Bacteria
90/88
85/69
FtsZ1 Archaea
94/81
78/63
FtsZ2 Archaea
98/97
An Naneq41615257
96/98
98/100
FtsZ Thaumarchaea
1
2
3
4
5
6
7
8
9
0.5

Constrained tree analysis of alternative tree topologies of the tubulin/FtsZ family tree

	Unconstrained tree			Constrained tree		
constraint #	ELW	AU	Likelihood	ELW	AU	Likelihood
1	0.950	0.9502	-20625.834	0.050	0.0498	-20649.030
2	0.963	0.9678	-20625.834	0.037	0.0322	-20638.978
3	0.837	0.8413	-20625.834	0.163	0.1587	-20634.967
4	0.867	0.8801	-20625.834	0.133	0.1199	-20631.958
5	0.830	0.8473	-20625.834	0.170	0.1527	-20632.346
6	0.862	0.8670	-20625.834	0.138	0.1330	-20642.431
7	0.897	0.9083	-20625.834	0.103	0.0917	-20633.350
8	0.782	0.7835	-20625.834	0.218	0.2165	-20637.708
9	0.718	0.7532	-20625.834	0.282	0.2468	-20627.738

The numbers of the constrained trees correspond to the alternative positions of the artubulin branch indicated on the tree above. Significant p-values according to AU are shown in red.
