# Supplementary material for: Archaeal origin of tubulin
Source: Biol Direct. 2012 Mar 29;7:10. doi: 10.1186/1745-6150-7-10 (PMC3349469; doi:10.1186/1745-6150-7-10)
Supplement: Additional file 4 — Additional phylogenetic trees constructed using the RAxML and TreeFinder methods. [file 1745-6150-7-10-S4.PDF]

Archaeal, Bacterial and Eukaryotic tubulins and FtsZ proteins (as in Fig. 2A)

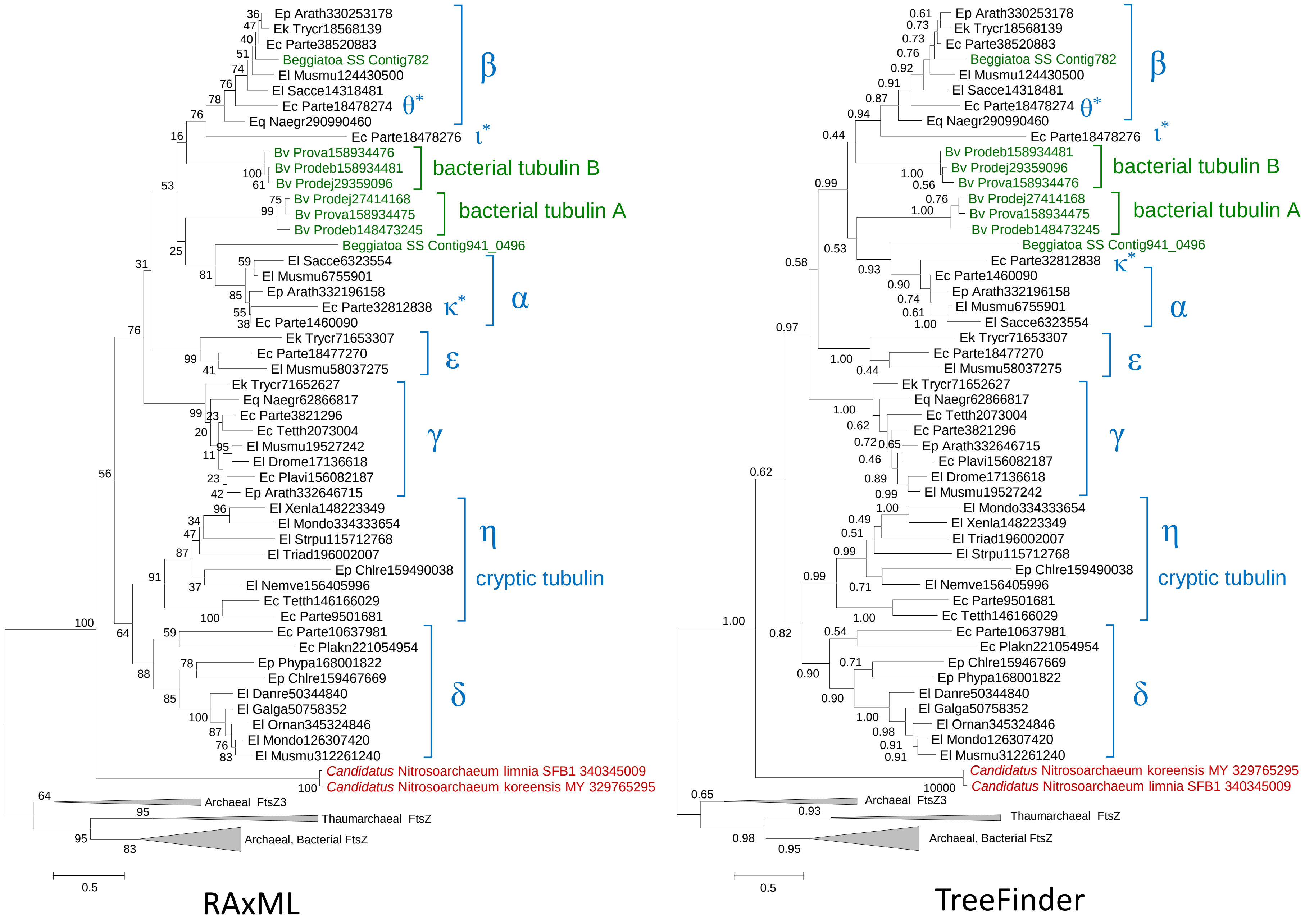

Bacterial and Eukaryotic tubulins

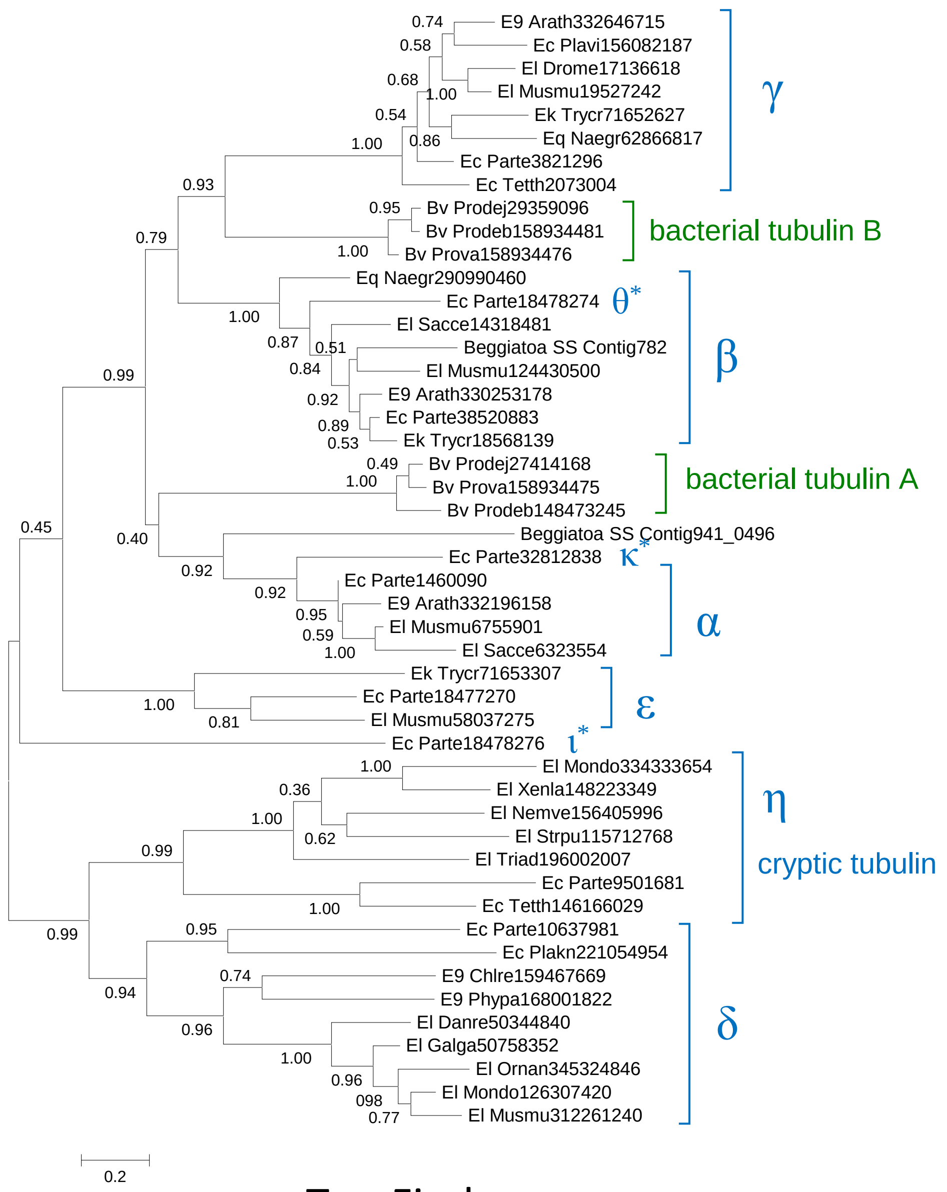

TreeFinder
